# Understanding the ontogeny and succession of *Bacillus velezensis* and *B. subtilis* subsp. *subtilis* by focusing on kimchi fermentation

**DOI:** 10.1038/s41598-018-25514-5

**Published:** 2018-05-04

**Authors:** Min Seok Cho, Yong Ju Jin, Bo Kyoung Kang, Yu Kyoung Park, ChangKug Kim, Dong Suk Park

**Affiliations:** Department of Agricultural Biotechnology, National Institute of Agricultural Sciences, Rural Development Administration, Jeonju, 54874 Republic of Korea

## Abstract

*Bacillus subtilis* and *B. velezensis* are frequently isolated from various niches, including fermented foods, water, and soil. Within the *Bacillus subtilis* group, *B. velezensis* and *B. subtilis* subsp. *subtilis* have received significant attention as biological resources for biotechnology-associated industries. Nevertheless, radical solutions are urgently needed to identify microbes during their ecological succession to accurately confirm their action at the species or subspecies level in diverse environments, such as fermented materials. Thus, in this study, previously published genome data of the *B. subtilis* group were compared to exploit species- or subspecies-specific genes for use as improved qPCR targets to detect *B. velezensis* and *B. subtilis* subsp. *subtilis* in kimchi samples. *In silico* analyses of the selected genes and designed primer sequences, in conjunction with SYBR Green real-time PCR, confirmed the robustness of this newly developed assay. Consequently, this study will allow for new insights into the ontogeny and succession of *B. velezensis* and *B. subtilis* subsp. *subtilis* in various niches. Interestingly, in white kimchi without red pepper powder, neither *B. subtilis* subsp. *subtilis* nor *B. velezensis* was detected.

## Introduction

*Bacillus* species are ubiquitous, endospore-forming, gram-positive bacteria that are of high economic importance due to their specific characteristics, such as their ability to colonize plants; to produce spores, biofilms and antibiotics; and to induce the synthesis of plant hormones^1,2^. Among *Bacillus* species, *B. subtilis* and *B. velezensis* have received considerable attention as biological resources in the food industry because they are accepted as safe, and their antagonistic activity may reflect competitive pressure in natural environments, leading to microbiota selection. Moreover, for *B. subtilis*, each subspecies presents different biological properties due to differences in metabolite synthesis. For example, *B. subtilis* subsp. *subtilis* strains were reported to synthesize either the lipopeptide surfactin, which has anti-*Listeria* activity, or surfactin and a novel bacteriocin, which have antibacterial properties. *B. velezensis* strains are potential protective starter cultures for the production of alkaline fermented foods, such as bikalga^3^. *B. velezensis* (its heterotypic synonyms include *B. methylotrophicus*, *B. amyloliquefaciens* subsp. *plantarum*, and *B. oryzicola*) and its sister species *B. subtilis* compose an evolutionarily small but physiologically relevant group of bacteria that includes strains isolated from diverse habitats^1,4–6^.

In general, the detection, quantitation, and identification of bacteria, including those in the genus *Bacillus*, are performed using various approaches, such as phenotypic, biochemical and immunological assays and molecular methods. However, when distinguishing between closely related species, these techniques have poor resolution and often result in the misclassification and misidentification of strains^7,8^. In particular, while the relative abundance of the genus *Bacillus* has been described for several types of kimchi using pyrosequencing, few data at the species level have been reported^9–14^.

For many years, 16S rRNA genes have been used to describe the microbiota composition of various environments. However, this analysis primarily identifies the relative abundances and diversity of bacteria and archaea in a sample. For the genus *Bacillus*, molecular analyses based on sequencing the 16S rRNA gene have been utilized as the primary tool for taxonomic assignment and phylogenetic tree construction of *Bacillus* species, including *B. subtilis*, *B. licheniformis*, and *B. velezensis*^4,5,15^.

However, species identification based on ribosomal RNA (rRNA) operon sequences, specifically the 16S rRNA genes, fails to distinguish one species from another, as their sequences have no significant differences. Thus, the use of these assays has significant drawbacks in the identification and detection of *Bacillus* groups, because they also detect other *Bacillus* species or subspecies. These technical limitations have become a significant obstacle that has prevented the elucidation of the microbial communities of various assayed samples, such as food or soil samples, even though pyrosequencing methods can provide insight into understanding the overall microbial composition of a particular niche^8,13,16–18^.

In the food industry, research has primarily focused on crucial bacteria, such as lactic acid bacteria (LAB) and bacilli, which play prominent roles in food fermentation processes and are thus of both scientific and industrial interest in food microbiology^19,20^. However, determining detection specificity, which can be influenced both by the uniqueness of DNA sequences within targeted bacterial genomes of interest and by the accurate annealing of primers or probes to their targets, is both critical for any PCR detection approach^17,21^ and urgently required.

The number of sequenced genomes has continued to increase with rapid advances in next-generation sequencing technology, allowing genes to be identified in the genome sequences of *Bacillus* strains that are unique to a taxon or a group of taxa^22^. This achievement, accompanied with the availability of various bioinformatics tools, has led to the advent of higher-fidelity, faster, and more cost-effective techniques for identifying *Bacillus* species in diverse environments. However, notwithstanding the present-day scientific advancements made using *Bacillus* strains in microbial industries, currently available solutions for identifying, detecting and quantifying a targeted *Bacillus* species or subspecies remain mostly limited, as is the case for other bacteria^5^.

Consequently, in this study, the bacterial genome sequences available in the NCBI GenBank ftp site (ftp://ftp.ncbi.nlm.nih.gov/genomes/genbank/bacteria/) were downloaded and compared to identify genes unique to *B. subtilis* subsp. *subtilis* or *B. velezensis* using a combination of bioinformatic tools^23,24^*. In silico* analyses of the selected genes and designed primer sequences, in conjunction with SYBR Green real-time PCR, confirmed that this newly improved PCR assay is able to precisely identify and quantify the two most prominent *Bacillus* species (*B. subtilis* subsp. *subtilis* and *B. velezensis*) at the species or subspecies level during kimchi fermentation, which is necessary for their use in food biotechnology.

Our findings demonstrated that this genome-based approach is advantageous for the accurate identification, detection and quantification of each targeted species in various environments. Interestingly, *B. subtilis* subsp. *subtilis* and *B. velezensis* were not detected in white kimchi without red pepper powder. However, the density of *B. subtilis* subsp. *subtilis* and *B. velezensis* was not particularly high in red pepper kimchi.

## Results

### *In silico* assay evaluation and PCR confirmation

The oligonucleotide primers and genes (Table 1) selected from two *Bacillus* species, i.e., *B. subtilis* subsp. *subtilis* or *B. velezensis*, were evaluated and confirmed via a combination of bioinformatic tools^23,24^.

**Table 1:** Primer sequences, their targets, and the annealing temperatures used in *Bacillus velezensis* and *B. subtilis* subsp*. subtilis* PCR screens.

Primer	Oligonucleotide sequence (5′-3′)	Annealing (°C)	Amplicon (bp)	Target gene (GenBank Accession No.)	Reference
Bam249F	GTCCGGGGGCATTGGCTGAG	67	249	putative hydrolase (NC020410.1)	This study
Bam249R	CCCCCTGCACATAGACGGACTGA				
BS310F	GGCCTATTGAACACCCTGATTTA	67	310	LysR family transcriptional regulator (NC020832.1)	This study
BS310R	CGGATGCGGCCTTCTTTTTC				

In *B. velezensis*, the BLASTn searches revealed no substantial matches to the recognized reference sequences of other *Bacillus* species. The results of the BLASTx searches, which used the predicted protein sequence of our putative hydrolase gene, showed that the most similar protein was a *Bacillus nakamurai* protein [identity = 61%, score = 564 bits (1,454), and expected = 0.0].

For *B. subtilis* subsp. *subtilis*, the BLASTn searches yielded no solid match to any of the other identified *Bacillus* reference sequences. The results of the BLASTx searches, which used the predicted protein sequence of our Ly*s*R family transcriptional regulator, showed that the most similar protein was from *Brevibacterium halotolerans* [identity = 94%, score = 413 bits (1,061), and expected = 2e-144].

**Figure 1:**
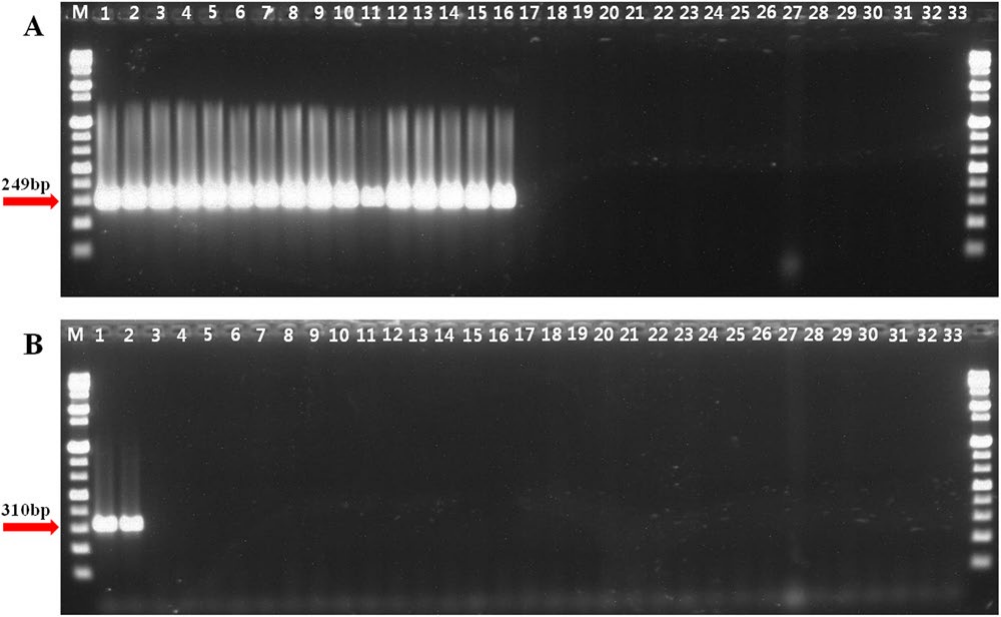
Specific PCR amplification of *Bacillus velezensis* (**A**) and *B. subtilis* subsp. *subtilis* (**B**) with the Bam249F/R and BS310F/R primer sets. (**A**) *B. velezensis*. Lane M is the size marker (1 kb DNA plus ladder; Gibco BRL). Lanes 1–16 are *B. velezensis* strains. Lanes 17–32 are strains of other *Bacillus* species, as listed in Table 2, and lane 33 is the negative control (distilled water). (**B**) *B. subtilis* subsp. *subtilis*. Lane M is the size marker (1 kb DNA plus ladder; Gibco BRL). Lanes 1–6 are *B. subtilis* strains. Lanes 7–32 are strains of other *Bacillus* species, as listed in Table 3 and lane 33 is the negative control (distilled water).

Specificity tests were performed using conventional PCR techniques for each species or subspecies primer set against DNA samples from various *Bacillus* strains, including the type strain of each targeted species or subspecies (Tables 2 and 3). Each expected specific PCR product was confirmed for each PCR assay, all of which were performed using genomic DNA from *B. velezensis* and *B. subtilis* subsp. *subtilis* strains (Fig. 1). Incidentally, the *B. subtilis* strains 10113, 10114, and 11994 from the Korean Agricultural Culture Collection (KACC) (Tables 2 and 3) were identified as *B. velezensis*, as shown in Fig. 1. Taken together, the results of the *in silico* evaluation and PCR confirmation showed that the PCR assay reliably identified *B. velezensis* and *B. subtilis* subsp. *subtilis*.

**Table 2:** Bacterial strains used in the PCR specificity test for *Bacillus velezensis*.

No.	Bacterial strains (Subjective synonym)	Source^a^	Host	This study^b^
1	*Bacillus amyloliquefaciens (Bacillus velezensis)*	LMG 12331	N.D.	+
2	*Bacillus amyloliquefaciens*	KACC 10116^T^	Takamine bacterial amylase concentrate	+
3	*Bacillus amyloliquefaciens*	KACC 17029	Soil	+
4	*Bacillus amyloliquefaciens*	KACC 17030	Soil	+
5	*Bacillus amyloliquefaciens*	KACC 17031	Soil	+
6	*Bacillus amyloliquefaciens*	KACC 17032	Soil	+
7	Bacillus amyloliquefaciens subsp. amyloliquefaciens	LMG 12325	N.D.	+
8	Bacillus amyloliquefaciens subsp. amyloliquefaciens	LMG 12326	N.D.	+
9	Bacillus amyloliquefaciens subsp. plantarum	LMG 26770^T^	N.D.	+
10	Bacillus amyloliquefaciens subsp. plantarum	LMG 12384	N.D.	+
11	Bacillus amyloliquefaciens subsp. plantarum	LMG 17599	N.D.	+
12	Bacillus siamensis	KACC 15859	Gochujang	+
13	*Bacillus subtilis*	KACC 10113	Soil	+
14	*Bacillus subtilis*	KACC 10114	Adenine and phenylalanine-requiring mutant	+
15	*Bacillus subtilis*	KACC 11994	Rhizosphere of Brassica napus	+
16	Bacillus methylotrophicus	LMG 27586^T^	N.D.	+
17	*Bacillus subtilis* subsp*. subtilis*	LMG 7135^T^	N.D.	−
18	*Bacillus subtilis*	KACC 10111	N.D.	−
19	Bacillus subtilis subsp. spizizenii	LMG 19156^T^	N.D.	−
20	Bacillus licheniformis	LMG 12363^T^	N.D.	−
21	Bacillus pumilus	LMG 18928^T^	N.D.	−
22	Bacillus sonorensis	LMG 21636^T^	N.D.	−
23	Bacillus vallismortis	LMG 18725^T^	N.D.	−
24	Bacillus atrophaeus	KACC 12090^T^	Soil	−
25	*Bacillus alveayuensis*	KACC 13323^T^	Deep sea sediment of the Ayu Trough (4000 m below sea level) in the western Pacific Ocean	−
26	*Bacillus pocheonensis*	KACC 14006^T^	Soil, ginseng field	−
27	*Bacillus kribbensis*	KACC 14005^T^	Soil	−
28	*Bacillus circulans*	KACC 14392^T^	Soil	−
29	*Bacillus firmus*	KACC 10897^T^	N.D.	−
30	*Bacillus jeotgali*	KACC 17399^T^	Jeotgal	−
31	*Bacillus niabensis*	KACC 11279^T^	N.D.	−
32	*Bacillus litoralis*	KACC 12148^T^	Sea water	−

^a^Superscript “T” indicates type strain.

^b^Conventional or real-time assay; + and − indicate species detected or not detected, respectively.

^c^N.D., Not determine.

**Table 3:** Bacterial strains used in the PCR specificity test for *Bacillus subtilis* subsp. *subtilis*.

No.	Bacterial strains	Source^a^	Host	This study^b^
1	*Bacillus subtilis subsp. subtilis*	LMG 7135^T^	N.D.^c^	+
2	*Bacillus subtilis*	KACC 10111	N.D.	+
3	*Bacillus subtilis*	KACC 10113	Soil	−
4	*Bacillus subtilis*	KACC 10114	Adenine and phenylalanine-requiring mutant	−
5	*Bacillus subtilis*	KACC 11994	Rhizosphere of Brassica napus	−
6	Bacillus subtilis subsp. spizizenii	LMG 19156^T^	N.D.	−
7	*Bacillus amyloliquefaciens*	LMG 12331	N.D.	−
8	*Bacillus amyloliquefaciens*	KACC 10116^T^	Takamine bacterial amylase concentrate	−
9	*Bacillus amyloliquefaciens*	KACC 17029	Soil	−
10	*Bacillus amyloliquefaciens*	KACC 17030	Soil	−
11	*Bacillus amyloliquefaciens*	KACC 17031	Soil	−
12	*Bacillus amyloliquefaciens*	KACC 17032	Soil	−
13	Bacillus amyloliquefaciens subsp. amyloliquefaciens	LMG 12325	N.D.	−
14	Bacillus amyloliquefaciens subsp. amyloliquefaciens	LMG 12326	N.D.	−
15	Bacillus amyloliquefaciens subsp. plantarum	LMG 26770^T^	N.D.	−
16	Bacillus amyloliquefaciens subsp. plantarum	LMG 12384	N.D.	−
17	Bacillus amyloliquefaciens subsp. plantarum	LMG 17599	N.D.	−
18	Bacillus siamensis	KACC 15859	Gochujang	−
19	Bacillus methylotrophicus	LMG 27586^T^	N.D.	−
20	Bacillus licheniformis	LMG 12363^T^	N.D.	−
21	Bacillus pumilus	LMG 18928^T^	N.D.	−
22	Bacillus sonorensis	LMG 21636^T^	N.D.	−
23	Bacillus vallismortis	LMG 18725^T^	N.D.	−
24	Bacillus atrophaeus	KACC 12090^T^	Soil	−
25	*Bacillus alveayuensis*	KACC 13323^T^	Deep sea sediment of the Ayu Trough (4000 m below sea level) in the western Pacific Ocean	−
26	*Bacillus pocheonensis*	KACC 14006^T^	Soil, ginseng field	−
27	*Bacillus kribbensis*	KACC 14005^T^	Soil	−
28	*Bacillus circulans*	KACC 14392^T^	Soil	−
29	*Bacillus firmus*	KACC 10897^T^	N.D.	−
30	*Bacillus jeotgali*	KACC 17399^T^	Jeotgal	−
31	*Bacillus niabensis*	KACC 11279^T^	N.D.	−
32	*Bacillus litoralis*	KACC 12148^T^	Sea water	−

^a^Superscript “T” indicates type strain.

^b^Conventional or real-time assay; + and – indicate species detected or not detected, respectively.

^c^N.D., Not determine.

### Real-time PCR assay efficiency, LOD and LOQ

**Figure 2:**
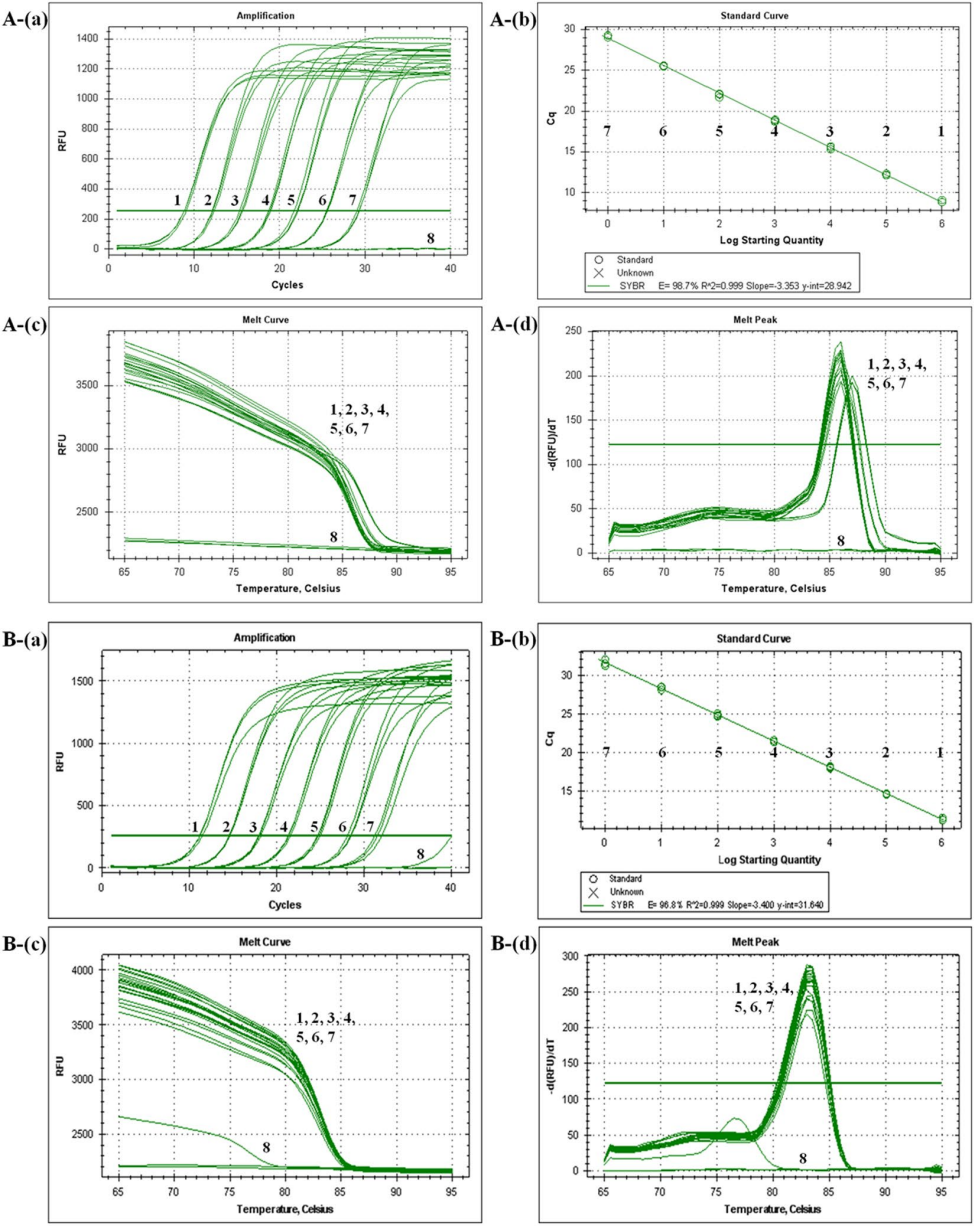
Specificity, melting peak and standard curve analysis of the SYBR Green qPCR assay with the Bam249F/R and BS310F/R primer sets. (**A**) *B. velezensis*. (a) Fluorescence intensity as a function of template concentration. For each assay, a series of 10-fold dilutions of cloned DNA (ranging from 1.42 × 10^3^ to 1.42 × 10^9^ copies/µl) was used as the template (1–7, sample dilutions). (b) Standard curve derived from the amplification plot. (c) Melting curve analysis (1–7, sample dilutions). (d) Melting peak analysis (1–7, sample dilutions). The amplified product derivatives of the relative fluorescence units [-d(RFU)/dT] were plotted as a function of temperature (amplified product, 86.0 °C). The large peak indicates the amplified product, while the small peak indicates the no-template control. (**B**) *B. subtilis* subsp. *subtilis*. (**a**) Fluorescence intensity as a function of template concentration. For each assay, a series of 10-fold dilutions of cloned DNA (ranging from 1.39 × 10^3^ to 1.39 × 10^9^ copies/µl) was used as the template (1–7, sample dilutions). (b) Standard curve derived from the amplification plot. (c) Melting curve analysis (1–7, sample dilutions). (d) Melting peak analysis (1–7, sample dilutions). The amplified product derivatives of the relative fluorescence units [-d(RFU)/dT] were plotted as a function of temperature (amplified product, 83.5 °C). The large peak indicates the amplified product, while the small peak indicates the no-template control.

Threshold cycle values (Ct) were considered the primary outcome variables for this study (Tables 4 and 5). The standard curves for the type strain of each targeted species, including *B. velezensis* and *B. subtilis* subsp. *subtilis*, were generated by charting the mean threshold cycle (Ct) (n = 3) based on logarithmic concentrations of genomic DNA (*B. velezensis*, from 5 to 5 × 10^−5^ ng/µl; and *B. subtilis* subsp. *subtilis*, from 5 to 5 × 10^−4^ ng/µl), cloned DNA (*B. velezensis*, from 1.42 × 10^9^ to 1.42 × 10^3^ copies/µl; and *B. subtilis* subsp. *subtilis*, from 1.39 × 10^9^ to 1.39 × 10^3^ copies/µl), and cell suspensions (*B. velezensis* and *B. subtilis* subsp. *subtilis* from 0.1 × 10^0^ to 0.1 × 10^−3^ OD_600_ units of cells per reaction). Limit of quantitation (LOQ) assays yielded linear responses and high correlation coefficients (*B. velezensis*, R^2^ = 0.999; and *B. subtilis* subsp. *subtilis*, R^2^ = 0.999). Standard curve analyses of the linear portions of the slopes for *B. velezensis* and *B. subtilis* subsp. *subtilis* produced coefficients of −3.353 and −3.400, which yielded PCR efficiencies of 98.7% and 96.8%, with y-intercept values of 28.942 and 31.640, respectively (Fig. 2). Melting analysis (curve, temperature, and peaks) of the real-time PCR reactions performed with the above *Bacillus* species provided reproducible melting temperatures (*B. velezensis*, 86.0 °C; and *B. subtilis* subsp. *subtilis*, 83.5 °C) and specific peaks (Fig. 2). The genomic DNA (*Bacillus velezensis*, R^2^ = 0.999, slope = −3.336; and *B. subtilis* subsp. *subtilis*, R^2^ = 0.997, slope = −3.456) and cell suspension (*B. velezensis*, R^2^ = 0.992, slope = −3.575; *B. subtilis* subsp. *subtilis*, R^2^ = 0.991, slope = −3.128) standard curves for each of the *Bacillus* species were linearly correlated with their respective Ct values, and their real-time PCR limits of detection (LOD) were 50–500 fg/μl of reaction mix and 0.1 × 10^−3^ OD_600_ units of cells per reaction (Tables 4 and 5). The SYBR Green real-time PCR assays for each *Bacillus* species presented excellent quantification and detection.

**Table 4:** Mean cycle threshold (CT) end-point fluorescence of 10-fold serial dilutions of *Bacillus velezensis* KACC 10116 cloned DNA, genomic DNA and a cell suspension determined with real-time PCR assay.

Cloned DNA	Genomic DNA			Cell suspension	
Weight/µl reaction mix	Ct ± SD^a^ (*n* = 3)	Weight/µl reaction mix	Ct ± SD (*n* = 3)	Cell density^b^	Ct ± SD (*n* = 3)
5 ng (1.42 × 10^9^ copies)	8.93 ± 0.16	5 ng	16.55 ± 0.15	OD_600_ = 0.1	22.22 ± 0.11
500 pg (1.42 × 10^8^ copies)	12.22 ± 0.12	500 pg	19.73 ± 0.07	OD_600_ = 0.01	25.34 ± 0.15
50 pg (1.42 × 10^7^ copies)	15.51 ± 0.19	50 pg	23.11 ± 0.05	OD_600_ = 0.001	28.76 ± 0.41
5 pg (1.42 × 10^6^ copies)	18.83 ± 0.14	5 pg	26.40 ± 0.05	OD_600_ = 0.0001	33.00 ± 0.41
500 fg (1.42 × 10^5^ copies)	21.96 ± 0.28	500 fg	29.91 ± 0.23	OD_600_ = 0.00001	N.D.^c^
50 fg (1.42 × 10^4^ copies)	25.53 ± 0.05	50 fg	33.14 ± 0.31	OD_600_ = 0.000001	N.D.
5 fg (1.42 × 10^3^ copies)	29.21 ± 0.09	5 fg	N.D.	OD_600_ = 0.0000001	N.D.

^a^SD, Three reactions standard deviation.

^b^OD = 600 nm.

^c^N.D., Not detected.

**Table 5:** Mean cycle threshold (CT) end-point fluorescence of 10-fold serial dilutions of *Bacillus subtilis* subsp. *subtilis* LMG 7135 cloned DNA, genomic DNA and a cell suspension determined with real-time PCR assay.

Cloned DNA		Genomic DNA		Cell suspension	
Weight/µl reaction mix	Ct ± SD^a^ (*n* = 3)	Weight/µl reaction mix	Ct ± SD (n = 3)	Cell density^b^	Ct ± SD (n = 3)
5 ng (1.39 × 10^9^ copies)	11.29 ± 0.18	5 ng	17.86 ± 0.07	OD_600_ = 0.1	20.65 ± 0.09
500 pg (1.39 × 10^8^ copies)	14.59 ± 0.06	500 pg	21.15 ± 0.19	OD_600_ = 0.01	23.30 ± 0.02
50 pg (1.39 × 10^7^ copies)	18.05 ± 0.12	50 pg	24.83 ± 0.03	OD_600_ = 0.001	26.74 ± 0.26
5 pg (1.39 × 10^6^ copies)	21.41 ± 0.14	5 pg	28.33 ± 0.48	OD_600_ = 0.0001	29.93 ± 0.72
500 fg (1.39 × 10^5^ copies)	24.81 ± 0.19	500 fg	31.55 ± 0.54	OD_600_ = 0.00001	N.D.^c^
50 fg (1.39 × 10^4^ copies)	28.34 ± 0.24	50 fg	N.D.	OD_600_ = 0.000001	N.D.
5 fg (1.39 × 10^3^ copies)	31.60 ± 0.45	5 fg	N.D.	OD_600_ = 0.0000001	N.D.

^a^SD, Three reactions standard deviation.

^b^OD = 600 nm.

^c^N.D., Not detected.

### Quantitative analysis of *Bacillus velezensis* and *B. subtilis* subsp. *subtilis* in kimchi samples using SYBR Green real-time PCR

Each kimchi sample was incubated at three different temperatures (4 °C, 15 °C, and 25 °C) and was subsequently tested for the presence and abundance of *B. velezensis* and *B. subtilis* subsp. *subtilis* using SYBR Green real-time PCR assays.

**Figure 3:**
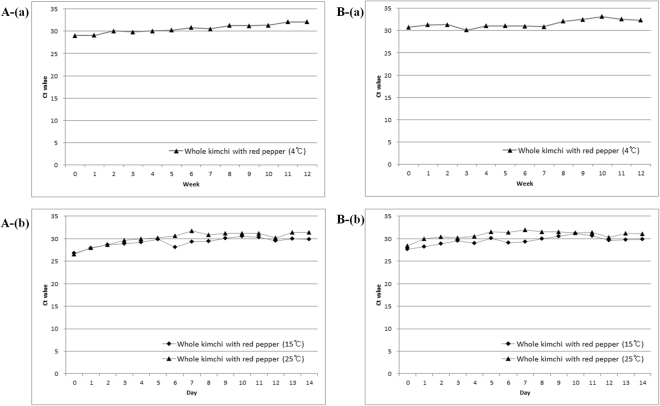
Changes in the real-time PCR Ct values during the quantification of *Bacillus velezensis* (**A**) and *B. subtilis* subsp. *subtilis* (**B**) using total DNA isolated from salted Chinese cabbage kimchi fermented at 4 °C (a), 15 °C and 25 °C (b).

*Bacillus velezensis* was not detected in any of the white kimchi samples (including the 4 °C, 15 °C, and 25 °C samples), but the corresponding samples from whole kimchi produced opposite results (Fig. 3). Generally, the 4 °C whole kimchi sample exhibited more delayed-fluorescence signals for the Ct value than those stored at 15 °C and 25 °C.

Similar to the findings for *B. velezensis*, all white kimchi samples (including the 4 °C, 15 °C, and 25 °C samples) tested negative for *B. subtilis* subsp. *subtilis*. In whole kimchi, the proportional changes of *B. subtilis* subsp. *subtilis* were similar to those observed for *B. velezensis* at 4 °C, 15 °C and 25 °C (Fig. 3). The samples collected from whole kimchi stored at 15 °C and 25 °C had lower Ct values than those stored at 4 °C between days 0 and 1. However, no significant difference in bacterial density was observed between the whole kimchi samples stored at 15 °C and those stored at 25 °C.

Therefore, the ontogeny of *B. velezensis* and *B. subtilis* subsp. *subtilis* was observed only in red pepper powder kimchi, regardless of the fermentation period. Furthermore, neither *B. velezensis* nor *B. subtilis* subsp. *subtilis* was a dominant species during the kimchi fermentation process.

## Discussion

*Bacillus* species continue to be leading bacterial workhorses in microbial industries. In particular, *B. velezensis*, which produces a natural antibiotic protein, an alpha amylase, a protease, and a restriction enzyme, and *B. subtilis*, one of the best understood prokaryotes, possess excellent genetic characteristics that have provided powerful tools and industrial resources to investigate targeted bacteria. However, although many approaches have been used to study the ecology and roles of various properties of *Bacillus* species, little is known about their ecological composition and succession in specific environments at the species or subspecies level.

For example, bacilli spores can persist for many years, but little is known about the dynamics of the germination, proliferation, movement, and sporulation for the spores of a given species in different niches, even though these bacteria are of increasing interest for use as biocontrol and probiotic agents^25,26^.

Recently, diverse *Bacillus* species have been identified in fermented foods, such as Korean cheonggukjang and kimchi, Chinese douchi, and Japanese natto. Of these species, the *B. subtilis* group contains the closely related taxa *B. subtilis* subsp. *subtilis*, *B. licheniformis*, *B. velezensis*, *B. atrophaeus*, *B. mojavensis*, *B. vallismortis*, and *B. subtilis* subsp. *spizizenii*^27^. In particular, *B. subtilis* subsp. *subtilis* and *B. velezensis* are the most frequently isolated bacilli from fermented foods or soil^5,28,29^.

*Bacillus subtilis* is a model beneficial bacterium with the ability to developmentally control many distinct cell types. As a significant producer of secreted enzymes*, B. subtilis*, together with *B. licheniformis*, is used at the industrial scale by biotechnology companies. *B. velezensis* is a primary growth-promoting rhizobacterium that has been reported to have enormous potential to support crop production in agricultural applications.

Nevertheless, further studies are required to determine the mechanisms of their beneficial activities. Although the various benefits of these species and their products have been demonstrated, no clear evidence describing the role of a particular bacterium has been presented. Furthermore, *B. methylotrophicus*, *B. amyloliquefaciens* subsp. *plantarum* and *B. oryzicola* were recently determined to be heterotypic synonyms of *B. velezensis*^4^.

In view of this finding, establishing reliable, efficient and specific molecular probes for the quantitative detection of a targeted bacterium in various niches is crucial, because such probes would enable the detection of individual species while providing an overall profile of the fluctuations in community structure in response to variations in time and temperature.

In early studies of *Bacillus subtilis* group communities, conventional approaches that used morphological or phenotypic identification methods of colonies grown on agar-solidified media were used. However, because these methodologies were unsatisfactory, DNA-based approaches began to be included to distinguish between *B. subtilis* group species and subspecies via 16S rRNA amplification. However, because 16S rRNA sequences exhibit over 98% similarity within this group, this method does not reliably distinguish or differentiate *Bacillus* species or subspecies^17,21^. Recently, whole genome shotgun sequencing was developed as an alternative approach to 16 rRNA amplicon sequencing and uses sequencing with random primers to sequence overlapping regions of a genome. However, this approach is more expensive and requires more extensive data analysis^30^.

Moreover, these techniques are not appropriate for determining the succession of a targeted bacillus at the species or subspecies level in various habitats.

Therefore, as stated above, the study of *Bacillus* group communities in fermented foods such as kimchi has been hindered by technical and cost-related issues.

Thus, the development of a reliable and effective procedure for quantitatively detecting specific bacilli used in commercial or scientific products is imperative. Fortunately, progress on the structural and functional genomics of a variety of *B. subtilis* group strains has provided insights into the microbial community dynamics of different environmental samples, revealing the parameters that influence variations in microbial communities. Determining the community structure of a microbiome and its key microbes has become easier due to the growing number of available microbial genome sequences. In particular, the availability of complete or draft *Bacillus* genome sequences provides an opportunity to improve their existing molecular detection and quantification tools by identifying new targets for more specific and sensitive detection.

To date, over 100 species of LAB and several yeast strains have been identified in kimchi, including *Weissella*, *Leuconostoc*, and *Lactobacillus* species. However, similar to other environments, there is little information regarding the succession or ontogeny of *Bacillus* species at the species level during kimchi fermentation, even though they are essential microorganisms in the food industry^13^.

In this study, we identified species or subspecies-specific genes using BLAST searches and designed primer sets to evaluate the population dynamics and ontogeny of *B. velezensis* and *B. subtilis* subsp. *subtilis*. The species-specific primer sets were designed using the whole genome sequences of *B. amyloliquefaciens* subsp. *plantarum* str. UCMB5036 (GenBank accession no. NC_020410.1) and *B. subtilis* subsp. *subtilis* str. BAB-1 (GenBank accession no. NC_020832.1) (Table 1). Selected primer sets, obtained through *in silico* analysis, in conjunction with SYBR Green real-time PCR, confirmed that this *de novo* qPCR assay is able to precisely identify and quantify the two most prominent *Bacillus* species, *B. subtilis* subsp. *subtilis* and *B. velezensis*, at the species or subspecies level^4,5^.

Recent reports on kimchi metabolites have indicated that the metabolite concentrations in red pepper powder kimchi are greater than those in kimchi without red pepper powder, likely because the addition of red pepper powder affects the metabolites in kimchi supernatants. In one case study of the food microbial community in kimchi, the percentage of *Weissella* was observed to be higher in red pepper powder kimchi than in kimchi without red pepper powder, whereas the abundances of *Leuconostoc* and *Lactobacillus* were lower in red pepper kimchi. In particular, the ontogeny of *W. cibaria* is significantly influenced by red pepper powder^12,13^.

Interestingly, as was observed for *W. cibaria*, the presence of *B. velezensis* and *B. subtilis* subsp. *subtilis* was only detected in whole kimchi with red pepper, regardless of the fermentation period. However, the abundances of these two microbes were not very high in red pepper kimchi during kimchi fermentation.

Accordingly, different types of kimchi and cheonggukjang were used to further determine whether red pepper powder is necessary and if it influences the ontogeny or density of *B. velezensis* and *B. subtilis* subsp. *subtilis* during kimchi fermentation. Both species were detected in chonggak kimchi (with red pepper powder) and red pepper cheonggukjang, a fermented soybean paste. However, contrary to our expectations, while they were both detected and identified in cheonggukjang without red pepper powder, they were not detected in watery kimchi without red pepper powder (data not shown).

Consequently, microbial metabolites present in kimchi are predicted to be influenced by red pepper powder, thereby influencing the ontogeny of *B. subtilis* subsp. *subtilis* and *B. velezensis* in whole kimchi, even though these are not the dominant species in the fermentative process. Moreover, the abundances of *B. subtilis* subsp. *subtilis* and *B. velezensis* were not significantly affected by the kimchi fermentation temperature conditions (4 °C, 15 °C and 25 °C). Changes in the proportion of *B. subtilis* subsp. *subtilis* were similar to those of *B. velezensis* at 4 °C, 15 °C and 25 °C (Fig. 3).

In conclusion, our results revealed that the *de novo* real-time PCR assay developed in this study has helped to overcome limitations in detecting *B. subtilis* subsp. *subtilis* and *B. velezensis* that were previously observed due to a lack of specificity or reproducibility in conventional and real-time PCR protocols. In addition, the good performance of the assay was also confirmed by quantifying and identifying these *Bacillus* strains, even with DNA isolated from kimchi samples.

Therefore, we believe that this approach can become the new gold standard method, as this evaluation will enable the rapid, culture-independent species- or subspecies-specific identification and quantification of these *Bacillus* strains in various industries, including fermented food.

## Methods

### Bacterial strains, culture conditions and genomic DNA isolation

A panel of 32 *Bacillus* reference strains was used for the specificity assay (Tables 2 and 3). Reference cultures were supplied by the Korean Agricultural Culture Collection (KACC) and the Belgian Coordinated Collections of Microorganisms (BCCM). The bacterial isolates were cultured on nutrient agar plates (BD Difco^TM^, USA) for 48 h at 30 °C under aerophilic conditions and were subcultured at least twice before use. Total genomic DNA was extracted from bacterial cells scraped from plates as previously described and was spectrophotometrically quantified (NanoDrop^®^ ND-1000 Spectrophotometer, NanoDrop Technologies, USA)^31^. All extracted genomic DNA samples were stored at -80 °C until their use in further experiments.

### DNA extraction from kimchi samples

Total DNA was extracted from kimchi samples using a Fast DNA Spin kit (MP bio, USA) according to the manufacturer’s instructions^31^. One microliter of each sample (5 ng/μl) was diluted individually for quantitative analysis.

In preparation of kimchi samples, two types of kimchi were obtained from a commercial factory in the Republic of Korea to investigate the ontogeny and succession of each targeted bacillus, *B. velezensis* and *B. subtilis* subsp. *subtilis*, during kimchi fermentation. Twenty-six batches of the two types of kimchi (13 batches each) were stored at 4 °C, while fifteen batches of each of the two types of kimchi stored at 15 °C and 25 °C were used, a total of 30 batches. The kimchi samples utilized in this experiment were obtained on the same day they were produced and were stored at different incubation temperatures (4 °C, 15 °C, and 25 °C) until sampling. As shown in Fig. 3, each sample was periodically filtered through sterilized coarse gauze (Daehan Co., Korea) during the fermentation period to collect the fluid portion. The filtrates were collected and then centrifuged (13,000 rpm for 10 min at 4 °C) to obtain the microorganisms in the kimchi.

### Candidate gene selection and oligonucleotide primer design for species- and subspecies-specific PCR assays

The comparative genomic analysis method was used to integrate the computational steps, which improved the candidate gene selection pipeline described by Chen and Lang via computational clustering^23,24^. The whole genome sequences (FASTA format) from *B. amyloliquefaciens* subsp. *plantarum* str. UCMB5036, *B. subtilis* subsp. *subtilis* str. BAB-1 and the other *Bacillus* species strains were downloaded from ftp://ftp.ncbi.nlm.nih.gov/genomes/bacteria/4,5. From this gene selection pipeline, target candidate genes sharing no significant homology with other *B. subtilis* group strains were designated PCR targets. The specific primers used for *B. velezensis* and *B. subtilis* subsp. *subtilis* were generated using the DNASTAR Lasergene primerselect module (version 7.0) (Table 1). Primer synthesis was performed by the Bioneer Corporation (Daejeon, Korea). Each primer set amplifies a specific DNA fragment from only the targeted species or subspecies. The nucleotide sequences of each primer set were assessed for their specificity via NCBI BLAST modules, including BLASTn and BLASTx (https://blast.ncbi.nlm.nih.gov/Blast.cgi).

### Species - and subspecies-specific PCR conditions

All conventional PCR reactions were performed in a total volume of 25 μl (1 × buffer, 0.2 mM of each dNTP, 4.0 mM MgCl_2_) with 1.25 U of GoTaq^®^ Flexi DNA polymerase (Promega, USA), 25 ng of template DNA and a 0.2 µM final concentration of each primer (Table 1). PCR was conducted using a PTC-225 thermocycler (MJ Research, Watertown, MA, USA) with the following steps: an initial denaturation period of 5 min at 95 °C, followed by 35 cycles of denaturation (1 min at 95 °C), annealing (30 s at 67 °C for both *B. velezensis* and *B. subtilis* subsp. *subtilis*), and extension (1 min at 72 °C), with a final extension period of 7 min at 72 °C. PCR products were subjected to electrophoresis on 1.5% (w/v) agarose gels in 1 × TBE buffer solution at 60 V for 2 h. All PCR products were stained using LoadingStar (DYNEBIO, Korea). Gel images were captured and documented using a VersaDoc 1000 gel imaging system (Bio-Rad Laboratories, USA).

### Quantitative PCR assay

Each qPCR reaction was performed in triplicate using the SYBR® Premix Ex Taq™ kit (Takara Bio, Japan). The total reaction mixture (20 µl) contained 10 µl of SYBR Green Mix, 1 µl of each primer (100 pM), 7 µl of RNase free water, and 1 µl of template. Samples were processed with a CFX96 real-time PCR system (Bio-Rad Laboratories, Hercules, CA, USA) using the following thermal cycling program: 95 °C for 10 min, followed by 40 cycles of 95 °C for 10 s, 55 °C for 30 s and 72 °C for 30 s. Melting curve analyses of PCR amplicons were initiated at 60 °C, with an incremental increase of 1 °C until a final temperature of 95 °C was reached. The standard curves of *B. velezensis* and *B. subtilis* subsp. *subtilis* were created by plotting the cycle threshold (CT) values of the qPCRs performed using a dilution series of genomic DNA, cloned DNA or a bacterial cell suspension. Absolute quantification and data analysis was performed automatically by the Bio-Rad CFX Manager^TM^ Version 1.6 suite. The copy number of the cloned DNA was calculated based on a previously described formula^12,31–33^.

## Electronic supplementary material

Supplementary Figure 1

## References

[1] Zhang N (2016). Comparative Genomic Analysis of *Bacillus amyloliquefaciens* and *Bacillus subtilis* Reveals Evolutional Traits for Adaptation to Plant-Associated Habitats. Front. Microbiol..

[2] Sabaté DC, Audisio MC (2013). Inhibitory activity of surfactin, produced by different *Bacillus subtilis* subsp. *subtilis* strains, against *Listeria monocytogenes* sensitive and bacteriocin-resistant strains. Microbiol. Res..

[3] Chaves-López C (2015). Diversity of food-borne *Bacillus* volatile compounds and influence on fungal growth. J. Appl. Microbiol..

[4] Dunlap CA, Kim SJ, Kwon SW, Rooney AP (2016). *Bacillus velezensis* is not a later heterotypic synonym of *Bacillus amyloliquefaciens*; *Bacillus methylotrophicus*, *Bacillus amyloliquefaciens* subsp. *plantarum* and ‘*Bacillus oryzicola’* are later heterotypic synonyms of *Bacillus velezensis* based on phylogenomics. Int. J. Syst. Evol. Microbiol..

[5] Fan B, Blom J, Klenk HP, Borriss R (2017). *Bacillus amyloliquefaciens*, *Bacillus velezensis*, *and Bacillus siamensis* Form an “Operational Group *B. amyloliquefaciens*” within the *B. subtilis* Species Complex. Front. Microbiol..

[6] Earl AM, Losick R, Kolter R (2007). *Bacillus subtilis* genome diversity. J. Bacteriol..

[7] Sangal V (2016). Next-generation systematics: An innovative approach to resolve the structure of complex prokaryotic taxa. Sci. Rep..

[8] Cho KM (2009). Novel multiplex PCR for the detection of lactic acid bacteria during kimchi fermentation. Mol. Cell Probes..

[9] Jeong SH, Jung JY, Lee SH, Jin HM, Jeon CO (2013). Microbial succession and metabolite changes during fermentation of dongchimi, traditional Korean watery kimchi. Int. J. Food Microbiol..

[10] Jung JY (2013). Metatranscriptomic analysis of lactic acid bacterial gene expression during kimchi fermentation. Int. J. Food Microbiol..

[11] Jeong SH, Lee SH, Jung JY, Choi EJ, Jeon CO (2013). Microbial succession and metabolite changes during long-term storage of Kimchi. J. Food Sci..

[12] Kang BK, Cho MS, Park DS (2016). Red pepper powder is a crucial factor that influences the ontogeny of *Weissella cibaria* during kimchi fermentation. Sci. Rep..

[13] Jung JY, Lee SH, Jeon CO (2014). Kimchi microflora: history, current status, and perspectives for industrial kimchi production. Appl. Microbiol. Biotechnol..

[14] Lee SH, Jung JY, Jeon CO (2015). Source Tracking and Succession of Kimchi Lactic Acid Bacteria during Fermentation. J. Food Sci..

[15] Huang CH, Chang MT, Huang L, Chu WS (2012). Development of a novel PCR assay based on the gyrase B gene for species identification of *Bacillus licheniformis*. Mol. Cell. Probes..

[16] Janda JM, Abbott SL (2007). 16S rRNA gene sequencing for bacterial identification in the diagnostic laboratory: pluses, perils, and pitfalls. J. Clin. Microbiol..

[17] Fernández-No IC (2011). Detection and quantification of spoilage and pathogenic *Bacillus cereus*, *Bacillus subtilis* and *Bacillus licheniformis* by real-time PCR. Food Microbiol..

[18] Johansson AH (2014). Studies of plant colonisation by closely related *Bacillus amyloliquefaciens* biocontrol agents using strain specific quantitative PCR assays. Antonie Van Leeuwenhoek..

[19] Park EJ (2012). Bacterial community analysis during fermentation of ten representative kinds of kimchi with barcoded pyrosequencing. Food Microbiol..

[20] Kim M, Chun J (2005). Bacterial community structure in kimchi, a Korean fermented vegetable food, as revealed by 16S rRNA gene analysis. Int. J. Food Microbiol..

[21] Fernández-No IC, Böhme K, Caamaño-Antelo S, Barros-Velázquez J, Calo-Mata P (2015). Identification of single nucleotide polymorphisms (SNPs) in the 16S rRNA gene of foodborne *Bacillus* spp. Food Microbiol..

[22] Belda E (2013). An updated metabolic view of the *Bacillus subtilis* 168 genome. Microbiology..

[23] Lang JM (2010). Genomics-based diagnostic marker development for *Xanthomonas oryzae* pv. *oryzae* and *X. oryzae* pv. *oryzicola*. Plant Dis..

[24] Chen J (2010). A real-time PCR method for the detection of *Salmonella enterica* from food using a target sequence identified by comparative genomic analysis. Int. J. Food Microbiol..

[25] Nicholson WL (2002). Bacterial endospores and their significance in stress resistance. Antonie Van Leeuwenhoek..

[26] Decker, A. R. & Ramamurthi, K. S. Cell Death Pathway That Monitors Spore Morphogenesis. *Trends Microbiol*. 10.1016/j.tim.2017.03.005 (2017). 10.1016/j.tim.2017.03.005 PMC5522370 28408070

[27] Ashe S, Maji UJ, Sen R, Mohanty S, Maiti NK (2014). Specific oligonucleotide primers for detection of endoglucanase positive *Bacillus subtilis* by PCR. 3 Biotech..

[28] Lee daE (2016). Metabolomic Profiles of *Aspergillus oryzae* and *Bacillus amyloliquefaciens* During Rice Koji Fermentation. Molecules..

[29] Ahn MJ, Ku HJ, Lee SH, Lee JH (2015). Characterization of a Novel Fibrinolytic Enzyme, *Bsf*A, from *Bacillus subtilis* ZA400 in Kimchi Reveals Its Pertinence to Thrombosis Treatment. J. Microbiol. Biotechnol..

[30] Ranjan R, Rani A, Metwally A, McGee HS, Perkins DL (2016). Analysis of the microbiome: Advantages of whole genome shotgun versus 16S amplicon sequencing. Biochem. Biophys. Res. Commun..

[31] Kang BK (2015). The influence of red pepper powder on the density of *Weissella koreensis* during kimchi fermentation. Sci. Rep..

[32] Yeates C, Gillings MR, Davison AD, Altavilla N, Veal DA (1998). Methods for microbial DNA extraction from soil for PCR amplification. Biol. Proced. Online..

[33] Whelan JA, Russel NB, Whelan MA (2003). A method for the absolute quantification of cDNA using real time PCR. J. Immunol. Meth..

