# Supplementary material for: Understanding the ontogeny and succession of Bacillus velezensis and B. subtilis subsp. subtilis by focusing on kimchi fermentation
Source: Sci Rep. 2018 May 4;8:7045. doi: 10.1038/s41598-018-25514-5 (PMC5935750; doi:10.1038/s41598-018-25514-5)

**Supplementary Information for**

**Understanding the ontogeny and succession of *Bacillus velezensis* and *B. subtilis* subsp. *subtilis* by focusing on kimchi fermentation**

**Min Seok Cho** ^*^**, Yong Ju Jin**^*^**, Bo Kyoung Kang, Yu Kyoung Park, ChangKug Kim and Dong Suk Park**

Department of Agricultural Biotechnology, National Institute of Agricultural Sciences, Rural Development Administration, Jeonju, 54874, Republic of Korea

^*^These authors contributed equally to this work.

Correspondence and requests for materials should be addressed to D.S.P. (email: dspark@rda.go.kr).

**Supplementary figure 1.** **Specific PCR amplification of *Bacillus velezensis* and *B. subtilis* subsp.** ***subtilis* with the Bam249F/R and BS310 primer sets.** Agarose gel electrophoresis original photo.


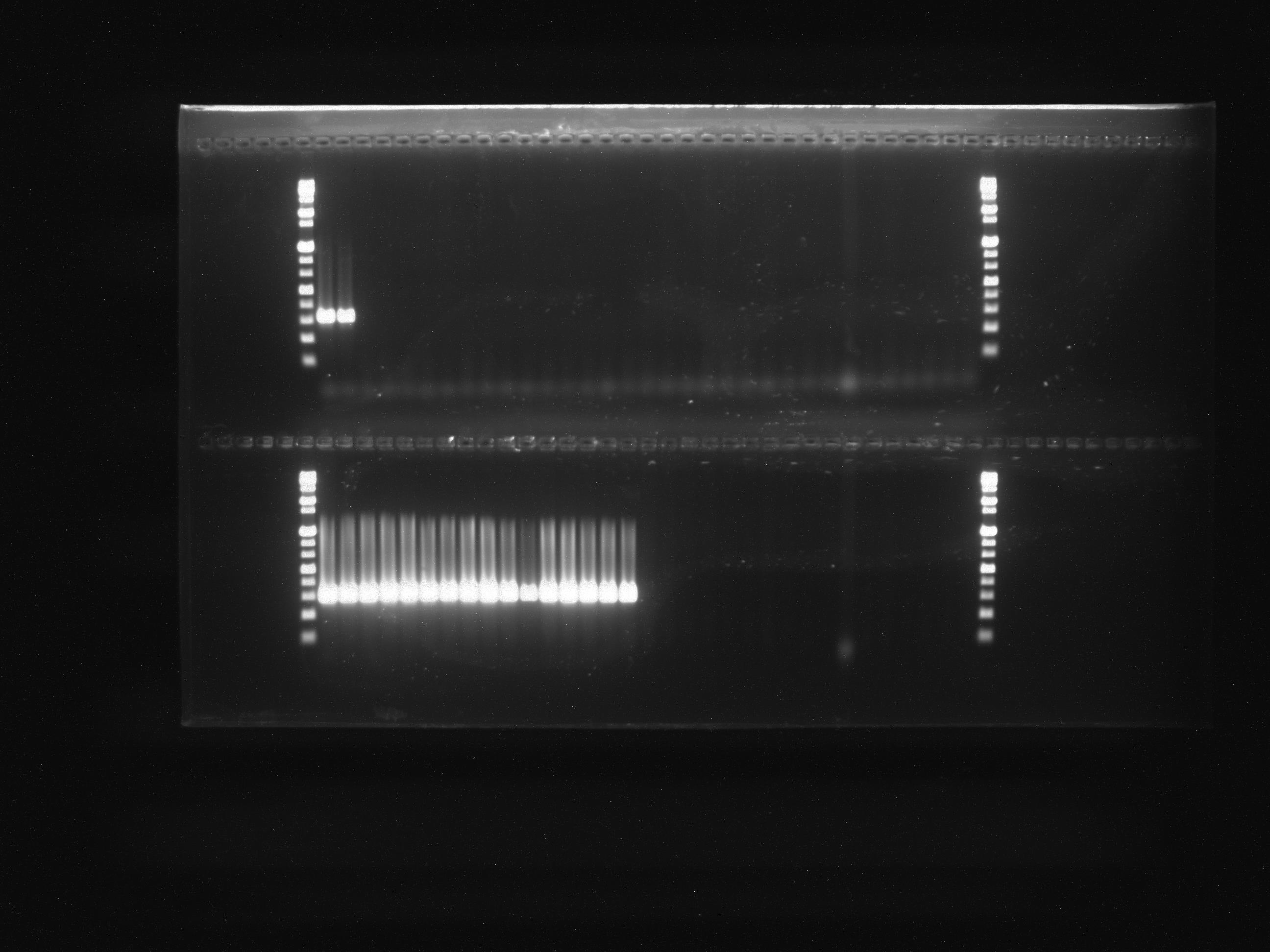

Supplement: Supplementary file 1 — Supplementary Figure 1 [file 41598_2018_25514_MOESM1_ESM.docx]
